# Shallow Landslide Susceptibility Mapping: A Comparison between Logistic Model Tree, Logistic Regression, Naïve Bayes Tree, Artificial Neural Network, and Support Vector Machine Algorithms

**DOI:** 10.3390/ijerph17082749

**Published:** 2020-04-16

**Authors:** Viet-Ha Nhu, Ataollah Shirzadi, Himan Shahabi, Sushant K. Singh, Nadhir Al-Ansari, John J. Clague, Abolfazl Jaafari, Wei Chen, Shaghayegh Miraki, Jie Dou, Chinh Luu, Krzysztof Górski, Binh Thai Pham, Huu Duy Nguyen, Baharin Bin Ahmad

**Affiliations:** 1 Geographic Information Science Research Group, Ton Duc Thang University, Ho Chi Minh City 758307, Vietnam; nhuvietha@tdtu.edu.vn; 2 Faculty of Environment and Labour Safety, Ton Duc Thang University, Ho Chi Minh City 758307, Vietnam; 3 Department of Rangeland and Watershed Management, Faculty of Natural Resources, University of Kurdistan, Sanandaj 66177-15175, Iran; a.shirzadi@uok.ac.ir; 4 Department of Geomorphology, Faculty of Natural Resources, University of Kurdistan, Sanandaj 66177-15175, Iran; h.shahabi@uok.ac.ir; 5 Board Member of Department of Zrebar Lake Environmental Research, Kurdistan Studies Institute, University of Kurdistan, Sanandaj 66177-15175, Iran; 6 Virtusa Corporation, 10 Marshall Street, Irvington, NJ 07111, USA; sushantorama@gmail.com; 7 Department of Civil, Environmental and Natural Resources Engineering, Lulea University of Technology, 971 87 Lulea, Sweden; 8 Department of Earth Sciences, Simon Fraser University, Burnaby, BC V5A 1S6, Canada; jclague@sfu.ca; 9 Research Institute of Forests and Rangelands, Agricultural Research, Education, and Extension Organization (AREEO), Tehran 13185-116, Iran; jaafari@rifr-ac.ir; 10 College of Geology & Environment, Xi’an University of Science and Technology, Xi’an 710054, China; chenwei0930@xust.edu.cn; 11 Key Laboratory of Coal Resources Exploration and Comprehensive Utilization, Ministry of Natural Resources, Xi’an 710021, China; 12 Department of Watershed Sciences Engineering, Faculty of Natural Resources, University of Agricultural Science and Natural Resources of Sari, Mazandaran 48181-68984, Iran; Shaghayegh.miraki@yahoo.com; 13 Department of Civil and Environmental Engineering, Nagaoka University of Technology, 1603-1, Kami-Tomioka, Nagaoka, Niigata 940-2188, Japan; douj888@gmail.com; 14 Faculty of Hydraulic Engineering, National University of Civil Engineering, Hanoi 112000, Vietnam; luuthidieuchinh@nuce.edu.vn; 15 Faculty of Mechanical Engineering, Kazimierz Pulaski University of Technology and Humanities in Radom, Chrobrego 45 Street, 26-200 Radom, Poland; krzysztof.gorski@uthrad.pl; 16 Institute of Research and Development, Duy Tan University, Da Nang 550000, Vietnam; 17 Faculty of Geography, VNU University of Science, 334 Nguyen Trai, Ha Noi 100000, Vietnam; huuduy151189@gmail.com; 18 Faculty of Built Environment and Surveying, Universiti Teknologi Malaysia (UTM), Johor Bahru 81310, Malaysia; baharinahmad@utm.my

**Keywords:** Shallow landslide, artificial intelligence, prediction accuracy, logistic model tree, goodness-of-fit, Iran

## Abstract

Shallow landslides damage buildings and other infrastructure, disrupt agriculture practices, and can cause social upheaval and loss of life. As a result, many scientists study the phenomenon, and some of them have focused on producing landslide susceptibility maps that can be used by land-use managers to reduce injury and damage. This paper contributes to this effort by comparing the power and effectiveness of five machine learning, benchmark algorithms—Logistic Model Tree, Logistic Regression, Naïve Bayes Tree, Artificial Neural Network, and Support Vector Machine—in creating a reliable shallow landslide susceptibility map for Bijar City in Kurdistan province, Iran. Twenty conditioning factors were applied to 111 shallow landslides and tested using the One-R attribute evaluation (ORAE) technique for modeling and validation processes. The performance of the models was assessed by statistical-based indexes including sensitivity, specificity, accuracy, mean absolute error (MAE), root mean square error (RMSE), and area under the receiver operatic characteristic curve (AUC). Results indicate that all the five machine learning models performed well for shallow landslide susceptibility assessment, but the Logistic Model Tree model (AUC = 0.932) had the highest goodness-of-fit and prediction accuracy, followed by the Logistic Regression (AUC = 0.932), Naïve Bayes Tree (AUC = 0.864), ANN (AUC = 0.860), and Support Vector Machine (AUC = 0.834) models. Therefore, we recommend the use of the Logistic Model Tree model in shallow landslide mapping programs in semi-arid regions to help decision makers, planners, land-use managers, and government agencies mitigate the hazard and risk.

## 1. Introduction

Landslides are a serious hazard in many parts of the world. According to a report by the World Bank, approximately 300 million people around the world live in landslide-prone areas [1,2] and economic losses from landslides amount to about USD 20 billion, with the largest losses incurred by the United States, Italy, Japan, India, China, and Germany [3,4,5].

Although landslides commonly can be attributed to natural (topographic, geological, geophysical, and hydrological) factors, Iran, like other countries, has experienced a large number of human-induced landslides in recent years due to ground modification and construction that has been driven by economic and population growth [5,6,7]. The country lies within a seismically active mountainous region, and large earthquakes trigger landslides in mountainous parts of the country, notably the Alborz and Zagros Mountains [8,9]. Nearly 2600 landslides were reported in Iran up to 2000 [10], and those in the 20th century alone are responsible for 30,000 deaths and nearly 60,000 injuries [11].

One strategy for reducing loss of life and damage from landslides is to prepare maps that identify areas vulnerable to landslides [12,13]. Landslide susceptibility may be defined as the likelihood that a landslide will occur in a given area or at a specific site [14]. Maps that show the propensity of an area to slope failure are termed “landslide susceptibility maps” [15,16]. These maps help land-use managers and other government officials to proactively reduce future losses from landslides [17].

In recent years, a variety of qualitative knowledge-driven and quantitative data-driven statistical and artificial intelligence (AI) techniques have been developed and used to predict landslides [18]. Although each method has its advantages and disadvantages, the Logistic Regression (LR) model method has been the first choice of most researchers [19]. The advantage of Logistic Regression is that variables can be discrete or any combination of types, and do not have to be normally distributed [20]. The LR uses a maximum likelihood estimation function to estimate the probability of an event occurring [21]. Developing accurate and robust models from environmental data has been a challenge for environmental scientists because of the “curse of multidimensionality,” i.e., environmental data are diverse in nature and come from a variety of sources, such as field surveys, air photo and satellite images, and historical records [17,18,22].

This problem has been addressed through the development and application of machine learning algorithms, which are able to handle large volumes of non-linear and complex data derived from different sources and reported at a variety of scales. These algorithms have been extensively used in natural hazard studies, for example: flooding [23,24,25,26,27,28,29,30,31,32,33], wildfire [34,35], dust storm [36], sinkhole formation [37], drought [38,39], earthquakes [40,41], gully erosion [42,43,44], land/ground subsidence [45,46], groundwater contamination [26,47,48,49,50,51], and landslides [17,52,53,54,55,56,57,58,59,60,61,62,63,64,65,66,67,68,69,70,71,72,73,74,75,76,77,78]. They can extract informative patterns in historical data to predict future events [79].

A wide variety of machine learning algorithms have been developed to overcome data challenges and develop accurate and robust landslide susceptibility prediction models. These algorithms extract related information patterns in historical data to forecast future events. Techniques that have been applied to develop landslide susceptibility maps include weight-of-evidence (WoE) [80], Logistic Regression (LR) [81,82,83], Bayesian Logistic Regression (BLR) [30,72], Artificial Neural Networks (ANN) [58,63,84,85,86], Evidential Belief Functions (EBF) [57,87], Fuzzy Logic Algorithm [88,89], Support Vector Machines (SVM) [52,90,91], Naïve Bayes Tree (NBT) [17,74,92,93], Alternating Decision Tree (ADTree) [45,54,69], Logistic Model Tree (LMT) [30,45,79], Kernel Logistic Regression (KLR) [94], Adaptive Neuro Fuzzy Inference System (ANFIS) [95], Gaussian process regression (GPR) [96], and Bagging Functional Tree (BFT) [97].

In particular, ensemble and hybrid machine learning techniques have provided promising results and have been widely used around the world in recent years [17,18,22,52,56,98,99]. Their base classifiers have good predictive ability and have been successful in predicting landslide-prone areas. For example, Naïve Bayes Tree (NBT), Logistic Model Tree (LMT), LR, Support Vector Machine (SVM), and Artificial Neural Network (ANN), the algorithms used in this study, have successfully identified landslide-susceptible areas in up to 90% of all cases [100,101,102].

The prediction accuracy of landslide susceptibility models depends on the geographical region, landslide conditioning factors (LCF), sample size, and on hyper parameter tuning [18,99]. As yet, there is no consensus as to which models are most appropriate for specific regions, hence it is necessary to use a variety of methods in each study area to determine the method with the highest predictive power. The main objective of this study is to spatially predict shallow landslides around Bijar city in eastern Kurdistan Province, Iran, using soft computing benchmark models, specifically LMT, LR, NBT, ANN, and SVM. The LR, ANN, and SVM algorithms are considered among some landslide researchers to be superior to other machine learning and conventional methods [22,103,104]. LMT is a decision tree algorithm that combines a decision tree and a Logistic Regression function in leaves. It has been used in many fields of environmental and natural hazard research, such as landslides, floods, gully erosion, sinkhole formation, land subsidence, and groundwater potential mapping [22,45,87,105,106]. In this study, we identify and rank the most important factors responsible for shallow landslides in the Bijar study area. We evaluate the reliability and predictive power of the five machine learning models based on sensitivity, specificity, accuracy, kappa, root mean square error (RMSE), area under the ROC curve (AUC), and Wilcoxon and Friedman statistical tests. Data processing was done using ArcGIS 10.3 (ESRI, Redlands, CA, USA), and the machine learning algorithms were modeled with WEKA 3.9 software (University of Waikato, Waikato, New Zealand).

## 2. Description of the Study Area

**Figure 1:**
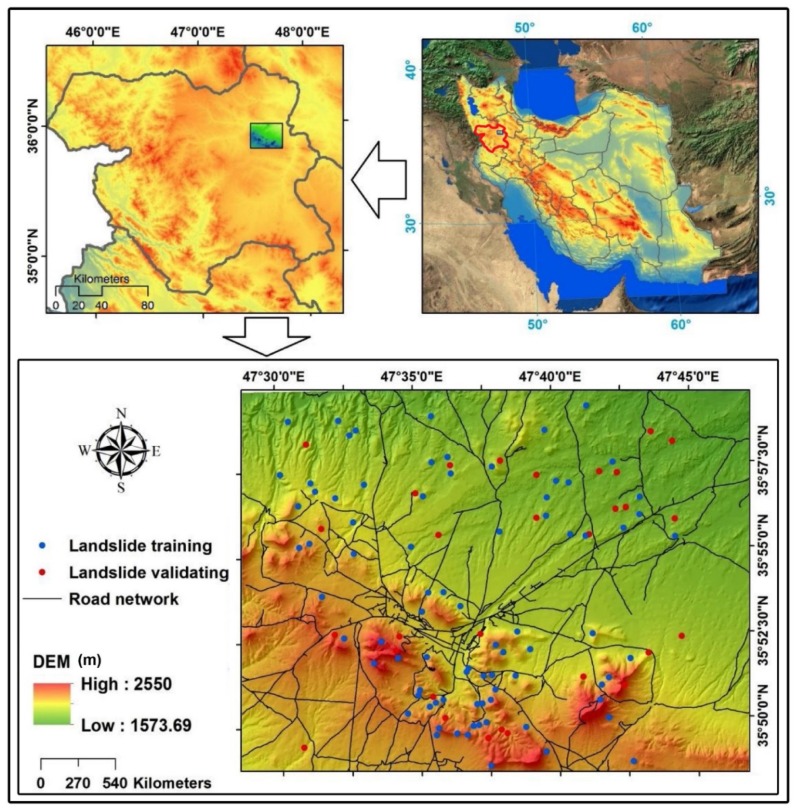
Location of shallow landslides in the study area. The blue circles denote landslides for training the algorithms, and the red circles denote landslides for validating the algorithms.

The study area (598 km^2^; 35°48′25″ N to 35°09′50″ N and 47°28′50″ E to 47°46′44″ E) is located around Bijar City in the eastern part of the Kurdistan Province in Iran (Figure 1). The regional climate is cool, with annual average temperatures ranging from 4.4 °C to 13.4 °C. Mean annual rainfall recorded between 1987 and 2010 at Bijar City was about 338 mm. Although annual precipitation is low, short-duration storms can produce large amounts of rain. Intensities of about 34 mm/h have a return period of about 20 years. The area is hilly, with elevations ranging from 250 to 1573 m asl (above sea level) and slopes up to 60°. There are four types of land cover in the Bijar region: (1) barren lands (0.07%), (2) cultivated lands (53.62%), (3) residential areas (1.26%), and (4) grasslands (45.05%). Geologically, 94% of the area is underlain by conglomerate, siltstone, shale, and marl, and 6% is underlain by volcanic rocks [17,65,69].

## 3. Data Preparation

### 3.1. Landslide Inventory Map

An accurate landslide inventory map is one of the prerequisite tools for achieving a successful landslide modeling prediction and must be prepared with care [107]. Some more important information can be derived from this inventory map, such as locations of occurred landslides, landslides type, frequencies of landslides, causes and triggers of landslides (i.e., earthquakes, intense rainfall, and rapid snowmelt) [65]. In this study, we obtained 111 landslide polygons from the Forests, Rangeland and Watershed Management Organization of Iran and checked them by examining aerial photographs (1:40,000 scale) and satellite images, and by inspection in the field. Field surveys showed that most of the landslides have resulted from human modification of slopes [84]. Most are shallow landslides (depths less than 3 m) and include slumps (70.60%), complex landslides (22.4%), and falls (6.3%) [86]. Minimum and maximum lengths of landslide are, respectively, 70 m and 280 m; the mean and median values are 37 m and 26 m. Landslide widths range from 7 to 293 m; mean and median values are 37 m and 63 m [65,69].

### 3.2. Landslide Conditioning Factors

In this study, we selected 20 landslide conditioning factors (LCF) based on the literature and data availability and partitioned them into five categories: topography (slope angle, slope aspect, elevation, curvature, profile curvature, plan curvature, and sediment transport index); hydrology (rainfall, solar radiation, sediment transport power (SPI), topographic wetness index (TWI), distance to rivers, and river density); geology (lithology, distance to fault, and fault density); land cover (land use and normalized difference vegetation index (NDVI)); and human-related factors (distance to road and road density). A digital elevation model (DEM) with a raster resolution of 28.5 m × 28.5 m was constructed from ASTER GDEM-1 satellite images taken in August 2005. The DEM was resampled to a raster resolution of 10 m to prepare data layers using the “Resample tool” in Arc GIS 10.3. The 20 LCM are briefly described below.

#### 3.2.1. Slope Angle

**Figure 2:**
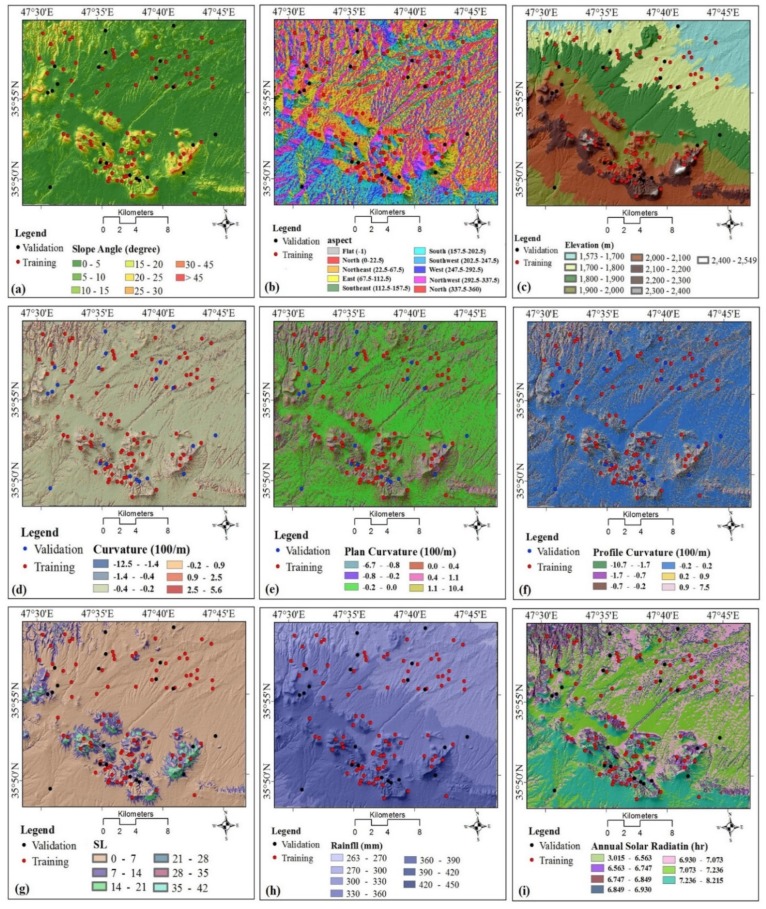

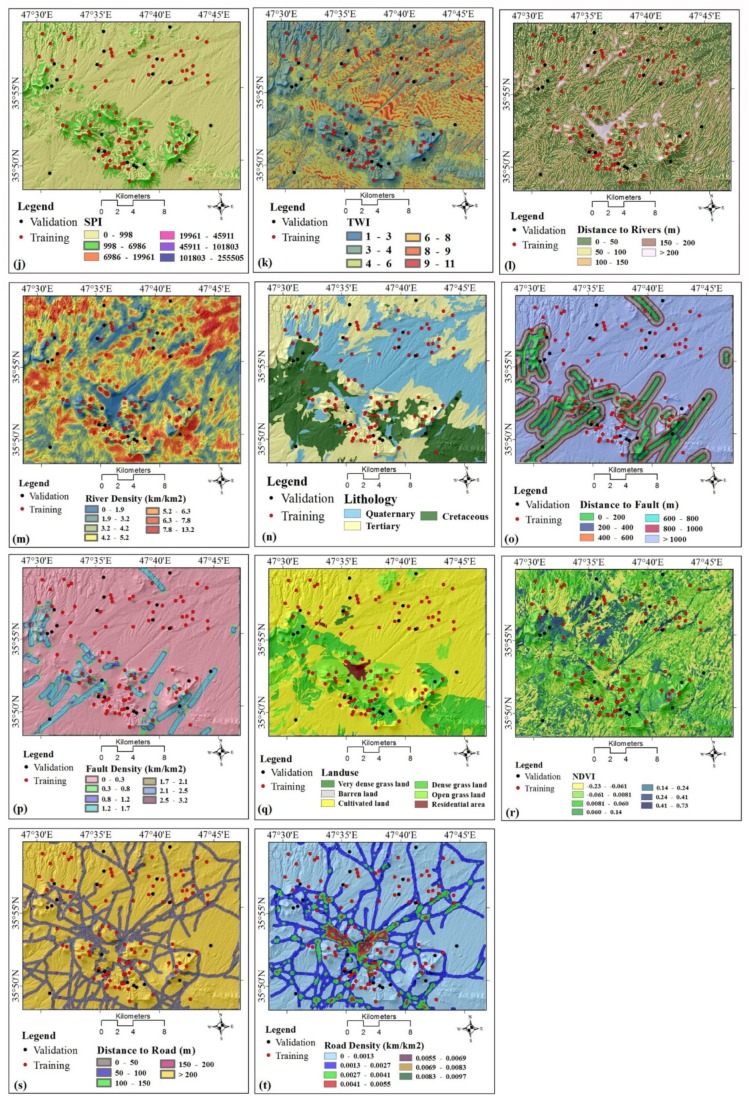
Landslide conditioning factors used in this study: (**a**) slope, (**b**) aspect, (**c**) elevation, (**d**) curvature, (**e**) plan curvature, (**f**) profile curvature, (**g**) slope length (SL), (**h**) rainfall, (**i**) annual solar radiation (**j**) stream power index (SPI), (**k**) topographic wetness index (TWI), (**l**) distance to rivers, (**m**) river density, (**n**) lithology, (**o**) distance to fault, (**p**) fault density, (**q**) land use, (**r**) normalized difference vegetation index (NDVI), (**s**) distance to road, (**t**) road density.

Slope is an expression of changes in elevations over distance and is expressed in this study in degrees. All other things being equal, steeper slopes are more susceptible to landslides, thus slope is an important conditioning factor in landslide susceptibility prediction modeling [5,17,18,108,109]. This conditioning factor was divided into eight classes using the manual classification method: (1) 0°–5°, (2) 5°–10°, (3)10°–15°, (4) 15°–20°, (5) 20°–25°, (6) 25°–30°, (7) 30°–45°, and (8) >45° (Figure 2a).

#### 3.2.2. Slope Aspect

Slope aspect is a measure of the cardinal direction of a slope, expressed relative to north (00) [110]. It has been shown to be related to the evapotranspiration in hilly areas and thus to be an important LCF [111,112]. In the present study, slope aspect was divided into nine classes: (1) flat, (2) north, (3) northeast, (4) east, (5) southeast, (6) south, (7) southwest, (8) west, and (9) northwest (Figure 2b).

#### 3.2.3. Elevation

The incidence and frequency of landslides may differ with elevation and thus can be an important LCD. Both temperature and precipitation affect soil moisture and commonly change with elevation. Lower elevations also may be preferentially used for roads, the construction of which might trigger landslides in hilly or mountainous areas [113]. Elevation may not have a fixed pattern, and it likely has different impacts on landslides depending on geology and the geographical region being studied [114]. Elevation was divided into nine classes using the manual classification method: (1) 1573–1700, (2) 1700–1800, (3) 1800–1900, (4) 1900–2000, (5) 2000–2100, (6) 2100–2200, (7) 2200–2300, (8) 2300–2400, and (9) >2400 m (Figure 2c).

#### 3.2.4. Curvature

The curvature of a slope can be concave, convex, or zero [109]. This LCM was divided into six classes using the natural break classification method: (1) [(−12.5)–(−1.4)]; (2) [(−1.4)–(−0.4)]; (3) [(−0.4)–(−0.2)]; (4) [(−0.2)–0.9]; (5) [0.9–2.5]; and (6) [2.5–15.6] (Figure 2d).

#### 3.2.5. Plan Curvature

Plan curvature is a measure of the concavity or convexity of a slope. It is used to analyze gradients between ridges and valleys. In this study, cells with concave curvature have positive values and cells with convex curvature have negative values. Slope erosion and water infiltration may relate to plan curvature; consequently, it is commonly used in developing landslide susceptibility prediction models [115,116]. We divided plan curvature into six classes using the natural break classification method: (1) [(−6.7)–(−0.8)]; (2) [(−0.8)–(−0.2)]; (3) [(−0.2)–0]; (4) [0–0.4]; (5) [0.4–1.1]; and (6) [1.1–10.4] (Figure 2e).

#### 3.2.6. Profile Curvature

Profile curvature is a measure of the concavity or convexity of the maximum slope, typically along stream channels. It can be positive, zero, or negative, depending on whether the surface is, respectively, upwardly concave, linear, or convex. As profile curvature influences water flow over the slope, it is considered to be one of the most important LCF in landslide susceptibility prediction models. We divided profile curvature into six classes using the natural break classification method: (1) [(−10.7)–(−1.7)], (2) [(−1.7)–(−0.7)], (3) [(−0.7)–(−0.2)], (4) [(−0.2)–0.2], (5) [0.2–0.9], and (6) [0.9–7.5] (Figure 2f).

#### 3.2.7. Slope Length

Slope length (SL) is the distance between the origin of overland flow and the point where runoff enters a defined channel [117]. It provides a measure of the erosive capability of overland flow [118]. We divided slope length into six classes using the natural break classification method: (1) 0–7; (2) 7–14; (3) 14–21; (4) 21–28; (5) 28–35; and (6) 35–42 (Figure 2g).

#### 3.2.8. Rainfall

The amount and intensity of rainfall is commonly positively correlated with landslide frequency, but the relationship depends strongly on topography. Rainfall on well drained, relatively flat terrain may have less impact on slope stability than it does in hilly areas [114]. We constructed a rainfall map based on mean annual rainfall over the period 1980–2016 based on records from nine rain gauge stations inside and outside the study area. Rainfall was divided into seven classes using the natural break classification method: (1) 263–270; (2) 270–300; (3) 300–330; (4) 330–360; (5) 360–390; (6) 390–420; and (7) 420–450 mm (Figure 2h).

#### 3.2.9. Annual Solar Radiation

Solar radiation directly affects evapotranspiration and is also influenced by topography. It may have an indirect impact on landslide susceptibility [113]. A layer for this conditioning factor was prepared using the “annual solar radiation” tool in ArcGIS 10.2, and then divided into seven classes using the natural break classification method: (1) 3.015–6.563, (2) 5.563–6.747, (3) 6.747–6.849, (4) 6.849–6.930, (5) 6.930–7.073, (6) 7.073–7.236, and (7) 7.236–8.215 h (Figure 2i).

#### 3.2.10. Stream Power Index

Stream power index (SPI) is a measure of the erosive capacity of flowing water and is a product of the slope gradient and catchment area [119], and is a good candidate for landslide susceptibility prediction model development. We derived SPI from the DEM in the SAGA software environment and then divided it into six groups: (1) 0–998; (2) 998–6986; (3) 6986–19,961; (4) 19,961–45,911; (5) 45,911–101,803; and (6) 101,803–255,505 (Figure 2j).

#### 3.2.11. Topographic Wetness Index

Topographic wetness index (TWI) is a measure of water accumulation degree at a site [118]. As TWI increases, landslide susceptibility may also increase. We calculated TWI from the DEM using SAGA software and then divided it into six groups using the natural break classification method: (1) 1–3; (2) 3–4; (3) 4–6; (4) 6–8; (5) 8–9; and (6) 9–11 (Figure 2k).

#### 3.2.12. Distance to Rivers

A river might undercut a side slope, increasing the likelihood of a landslide. We infer, however, that this is only likely to happen if the river borders a slope steeper than 15°. Thus, when constructing the distance-to-river map, we did not consider instances where rivers border slopes of less than 15°. We constructed the distance-to-river map with buffers around rivers using the Euclidean distance tool in ArcGIS 10.5, and then divided distances into five groups using the natural break classification method: (1) 0–50, (2) 50–100, (3) 100–150, (4) 150–200, and (5) >200 m (Figure 2l).

#### 3.2.13. River Density

Rivers density is positively related to the frequency of landslides in mountainous regions [120], in part through its effects on groundwater recharge. The river density layer was prepared using the line density tool in ArcGIS 10.2 and then divided into seven groups using the natural break classification methods: (1) 0–1.9, (2) 1.9–3.2, (3) 3.2–4.2, (4) 4.2–5.2, (5) 5.2–6.3, (6) 6.3–7.8, and (7) 7.8–13.2 km/km^2^ (Figure 2m).

#### 3.2.14. Lithology

Lithology affects soil porosity and permeability [121] and also rock strength. The lithology map of the study area was generated from a 1:100,000-scale geological map produced by the Geological Survey of Iran and verified through field work and air photo interpretation. We grouped geological units into three groups: Quaternary, Tertiary, and Cretaceous (Figure 2n).

#### 3.2.15. Distance to Faults

Many landslides are associated with faults due to the lower strength of rocks along these structures. We prepared the distance-to-fault layer from the geological map using Euclidean distance tool in ArcGIS 10.2; values range from 0 to 2000 m. We divided the LCF into five groups using the natural break classification method: (1) 0–200, (2) 200–400, (3) 400–600, (4) 600–800, (5) 800–1000 and (6) >1000 m (Figure 2o).

#### 3.2.16. Fault Density

Fault density provides an aerial measure of highly fractured, and thus weak, rocks [122]. The fault density layer was produced from the geological map using the line density tool in ArcGIS 10.2 and then divided into seven groups using the natural break classification method: (1) 0–0.3, (2) 0.3–0.8, (3) 0.8–1.2, (4) 1.2–1.7, (5) 1.7–2.1, (6) 2.1–2.5, and (7) 2.5–3.2 km/km^2^ (Figure 2p).

#### 3.2.17. Land Use

Land use is a significant factor for slope stability because development and utilization of the land affects infiltration, surface runoff, and vegetation [123]. The land-use layer in the present study was generated from Landsat 7 OLI sensor images. Six land-use types were identified: (1) very dense grassland, (2) barren land, (3) cultivated land, (4) dense grassland, (5) open grassland, and (6) residential area (Figure 2q).

#### 3.2.18. NDVI

The normalized difference vegetation index (NDVI) provides a measure of vegetation greenness and thus biomass. A change in vegetated areas might lead to slope failures [124]. We prepared the NDVI map using Landsat 8 OLI sensor images from Landsat 8 in ENVI5.1. NDVI values were divided into six categories: (1) [(−0.23)–(−0.061)], (2) [(−0.061)–(−0.0081)], (3) [(−0.0081)–(0.060)], (4) [(0.060)–0.14], (5) [0.14–0.24], (6) [0.24–0.41], and (7) [0.41–0.73] (Figure 2r).

#### 3.2.19. Distance to Roads

Road construction can increase the likelihood of landslides in hilly and mountainous areas by reducing the rock and sediment strength, steepening slopes, and creating roadside fills [125]. Only roads that undercut slopes steeper than 15° were included in the distance-to-road map. We added buffer zones to calculate distances from roads at five intervals: (1) 0–50, (2) 50–100, (3) 100–150, (4) 150–200, and (5) >200 m (Figure 2s).

#### 3.2.20. Road Density

Road density is the cumulative length of roads per unit area [126]. Most landslides in the study area are near roads, therefore road density provides a measure of the cumulative impacts of road construction on the occurrence of landslides [17]. The road density layer has seven categories: (1) 0–0.0013, (2) 0.0013–0.0027, (3) 0.0027–0.0041, (4) 0.0041–0.0055, (5) 0.0055–0.0069, (6) 0.0069–0.0083, and (7) 0.0083–0.0097 km/km^2^ (Figure 2t).

## 4. Methods

### 4.1. Naïve Bayes Tree

The Naïve Bayes Tree (NBT) model, which was first proposed by Kohavi [127], combines two classifiers: the ID3 decision tree, which is responsible for the classification process and splitting the tree, and Naïve Bayes. It offers several advantages over other machine learning models, specifically the ability to (1) represent knowledge, (2) manage complexity, (3) select candidate concepts, (4) process small datasets, and (5) minimize noise in training datasets [128]. The modeling and classification processes can be performed on even a small amount of data [111].

The first step in NBT modeling is to grow a tree based on the entropy (degree of disorder) feature selection method. If S is a set of the training dataset and |S| is the total number of conditioning factors, the factors can be grouped into n classes $S_{i}(i=1,2,\ldots ,n)$. ${\left|S\right|}_{i}$ is the number of conditioning factors belonging to the class $S_{i}$. The classification of S can be calculated based on the expected entropy as follows:

(1) $$\mathrm{Entropy}(S)=-\sum_{i=1}^{n}(\frac{\left|S_{i}\right|}{\left|S\right|})log_{2}[(\frac{\left|S_{i}\right|}{\left|S\right|})]$$

Consider attribute A, for example aspect, in set *S*. The expected entropy can be expressed as:

(2) $${\mathrm{Entropy}}_{A}\left(S\right)=-\sum_{i=1}^{n}\frac{\left|S_{i}\right|}{\left|S\right|}\times Info(S_{i})$$

The difference between Entropy(S) and ${Entropy}_{A}\left(S\right)$ is represented as the information gain (InfoGain):

(3) $$\mathrm{InfoGain}(A)=Entropy(S)-Entropy_{A}(S)$$

The information gain ratio (IGR) is calculated according to the following equation:

(4) $$GainRatio(A)=\frac{\mathrm{InfoGain}(A)}{SplitInfo(A)}=\frac{Entropy(S)-Entropy_{A}(S)}{-\sum_{i=1}^{n}(\frac{\left|S_{j}\right|}{\left|S\right|})\log_{2}[(\frac{\left|S_{j}\right|}{\left|S\right|})]}$$

Naïve Bayes is performed on the leaves of the tree after the tree is grown and split. It assumes the conditional independence among attributes $x_{1},x_{2},\ldots ,x_{n}$. Let $k_{i}(landslide\;and\;non-landslide)$ be an attribute of class set K. The a posteriori probability can be computed as follows:

(5) $$k_{NB}=\arg\max_{Z_{i}}PP(k_{i})\prod_{i=1}^{n}\frac{1}{\sqrt{2\pi \sigma }}e^{\frac{-{\left(d_{i}-\sigma \right)}^{2}}{2\sigma^{2}}}$$

where $PP(k_{i})$ denotes the a posteriori probability of the output class label $k_{i}(landslide\;and\;non-landslide)$, and *σ* and *ε* are, respectively, the mean and standard deviation of $d_{i}$.

### 4.2. Logistic Regression

Logistic Regression determines the relation of landslide occurrence and possible causative factors, and has been widely used in landslide susceptibility mapping [129,130]. It can be used when the dependent variable is binary or dichotomous. The dependent variable (Y) is the absence (0) or presence (1) of a landslide. The conditional probability that a landslide occurs is denoted by P(y=1|x). The logit of the LR model is transformed by the following equation:

(6) $$logit\left(y\right)=b_{0}+b_{1}x_{1}+b_{2}x_{2}+\ldots +b_{n}x_{n}$$

where $b_{0}$ is the intercept of the equation, and $b_{1}$, $b_{2}$, …,$\;b_{n}$ are the coefficients of independent variables $x_{1}$, $x_{2}$, …,$\;x_{n}$. The probability P(y=1|x) is computed in the LR model as follows:

(7) $$P(y=1|x)=\frac{1}{1+e^{-(}{}^{b_{0}+b_{1}x_{1}+b_{2}x_{2}+\ldots +b_{n}x_{n})}}$$

where e is the constant 2.718.

### 4.3. Logistic Model Tree

Logistic Model Tree combines the C4.5 algorithm [131] and Logistic Regression (LR) functions. The IGR technique is applied to split the tree into nodes and leaves, and the LogitBoost algorithm [132] is used to fit the logistics regression functions at a tree node. The C4.5 algorithm uses the entropy technique for feature selection because it is the fastest method for providing reliable classification accuracy [133]. The over-fitting problem, which is an important challenge in LMT modeling, is overcome using the CART algorithm, which prunes the tree for modeling the training dataset [129].

The IGR can be formulated as follows:

(8) $$\mathrm{Gain}\;\mathrm{ratio}\;(A)=\frac{\mathrm{gain}(A)}{\mathrm{split}\;\mathrm{info}(A)}$$

where gain(A) is the information after attribute A is selected as a test for classification of the training samples and split info(A) is the information generated when x training samples are categorized into n subsets [131].

In the next step, the LogitBoost algorithm performs additive Logistic Regression with least-squares fit for each class *Ci* (landslide or non-landslide) according to the following equation [134]:

(9) $$L_{c}(x)=\sum_{i=1}^{CF}\alpha_{i}x_{i}+\alpha_{0}$$

where $L_{c}(x)$ is the least-squares fit, and CF $\alpha_{i}$ are, respectively, the number of landslide conditioning factors and the coefficient of the *i*th element of vector *x*. The a posteriori probabilities in the leaves of the LMT are calculated using the linear Logistic Regression model [132]:

(10) $$p(c|x)=\frac{\exp(L_{c}(x))}{\sum_{c^{\prime }=1}^{c}\exp(L_{c^{\prime }}(x))}$$

where c is the number of landslide classes and *Lc(x)*, the least-squares fit, is transformed in such a way that $\sum_{c^{\prime }=1}^{c}L_{c}(x)=0$.

### 4.4. Support Vector Machine

**Figure 3:**
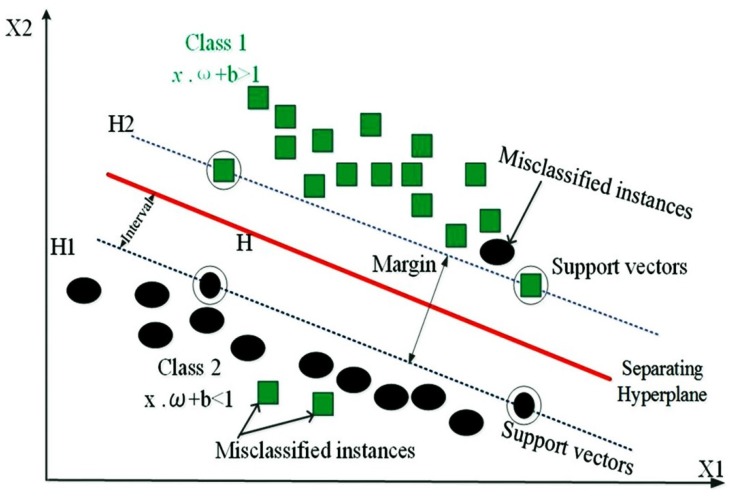
Illustration of the Support Vector Machines (SVM) method.

Support Vector Machine is a set of machine learning techniques based on the concept of an optimal separating hyperplane [135]. SVM finds the widest margin between two classes in feature space. A typical SVM model can be a two-class or multi-class model (combination of a chain of two-class SVMs), as shown in Figure 3. The two-class SVM is the most frequently used machine learning model [94,136,137]. During model performance, the separating hyperplane is one of the probable planes separating the two classes. SVM finds an optimal hyperplane by distinguishing the two classes, using the following equation [135]:

(11) $$\overset{}{\underset{w,b,\xi }{Min}}:\;\frac{1}{2}w^{T}w+c{\sum_{i=1}^{1}\xi }_{i}$$

subject to the following constraints:

(12) $$\begin{array}{l} y_{i}(w^{T}\phi (x_{i})+b)\geq 1-\xi_{i}\\ \;\xi_{i}\geq 0 \end{array}$$

where *w* is a coefficient vector, *b* is the offset of the hyperplane from the beginning, $\xi_{i}$ is the positive slack variable, and *c* (> 0) signifies the penalty parameters of the errors.

### 4.5. Artificial Neutral Network

ANNs are networks of processing neurons that operate the data and communicate with other components [138]. An advantage of ANNs is that they can use some a priori unknown information hidden in the data. In principle, they can be employed in linear or nonlinear models and single- or multi-layer networks. ANN is a very popular artificial intelligence method and has been extensively used in landslide susceptibility mapping and detection [85,139,140,141].

**Figure 4:**
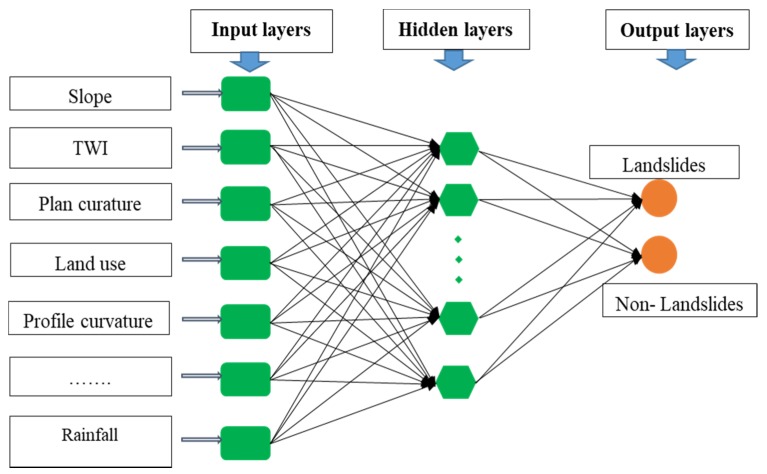
Flowchart of the Artificial Neutral Network (ANN) model.

Most ANNs comprise three sets of layers: input layer, hidden layers, and output layers (Figure 4). At computation nodes, each entering value is multiplied by the assembly weight. The yields are next summed with a neuron-specific constraint, called bias, that is used to scale the sum of the yields into a suitable range. Lastly, the computational node relates an activation function to the above sum, producing the node output. Weights and biases are computed by means of a non-linear optimization training procedure, which minimizes a learning function that conveys proximity between observations and ANN output.

Let it be known that *u = u_1_, u_2_, …, u_n_* denote n input neurons, and *v = v_1_, v_2_* denote output neurons. For the classification, the activation function used in hidden neurons is computed as:

(13) $$v=f\left(\sum_{i=1}^{n}\omega_{i}u_{i}+\beta_{}\right)$$

where $w_{ji}$ are connected weights between input neurons $u_{i}$ and output neurons *v*, and β is the bias.

### 4.6. Model Comparison and Validation

#### 4.6.1. Statistical Metrics

We used five statistical measures to evaluate the new proposed benchmark model and other soft computing models, namely sensitivity (SE), specificity (SP), accuracy (AC), mean absolute error (MAE), and root mean square error (RMSE). Sensitivity, specificity, and accuracy were calculated based on four possible consequences: true positive (TP), false positive (FP), true negative (TN), and false negative (FN). TP and FP are the numbers of landslide cells that are correctly categorized as, respectively, landslides and non-landslides. TN and FN are the numbers of landslide cells that are correctly categorized and incorrectly categorized as non-landslides. SE is the ratio of the number of correctly categorized landslide cells to the total number of predicted landslide cells. SP is the ratio of the number of incorrectly categorized landslide cells to the total predicted non-landslide cells. Accuracy is the ratio of the number of correctly categorized landslide cells to correctly categorized non-landslide cells. The RMSE index was used to evaluate the difference between observed and estimated data. The performance of landslide models is better when the values of sensitivity, specificity, and accuracy are high and the RMSE value is low. The formulas for these statistical measures are the following [142,143,144,145,146]:

(14) $$\mathrm{SE}=\frac{\mathrm{TP}}{\mathrm{TP}+\mathrm{FN}}$$

(15) $$\mathrm{SP}=\frac{\mathrm{TN}}{\mathrm{TN}+\mathrm{FP}}$$

(16) $$\mathrm{AC}=\frac{\mathrm{TP}+\mathrm{TN}}{\mathrm{TP}+\mathrm{TN}+\mathrm{FP}+\mathrm{FN}}$$

(17) $$MAE=\frac{1}{N}\sum_{i=1}^{N}\left|X_{predicted}-X_{actual}\right|$$

(18) $$RMSE=\sqrt{\frac{1}{n}}\sum_{i=1}^{n}(X_{predicted}-X_{actual}{)}^{2}$$

where $X_{predicted}$ and $X_{actual}$ are the predicted and real values of *X* in the training or testing dataset of the landslide susceptibility model, and n is the total number of samples in the training or testing dataset.

#### 4.6.2. ROC Curve and AUC Metric

The Receiver Operating Characteristic (ROC) is a standard tool for evaluating model performance [18,62,93]. ROC is displayed on a plot of sensitivity on the x-axis and 100-specificity on the y-axis. We used AUC (Area under the ROC curve) to show model performance [147]. We calculated the success rate, the prediction rate, and their AUCs. The mathematical basis and formula for this method are described in previous studies [64]. Sensitivity (i.e., detection probability) addresses the correct classification of observed landslides; if all observed landslides are correctly classified, the value is 1 [148]. In contrast, specificity (i.e., negative predictive value) addresses the correct classification of non-landslides; again, its value is 1 if all non-landslides are correctly classified. The ROC of the training dataset indicates the success rate and suitability of the model [49,149]. The ROC of the testing dataset gives the predictive success of the model and thus how good or poor it is as a predictor [94,150]. AUC values of <0.6, 0.6–0.7, 0.7–0.8, 0.8–0.9, and >0.9 indicate, respectively, poor, moderate, good, very good, and excellent model performance.

#### 4.6.3. Friedman and Wilcoxon Sign Rank Statistical Tests

We used the Freidman and Wilcoxon ranking tests to further evaluate the performance of the new proposed landslide model relative to the other models considered in this paper [93,151]. The probability of a hypothesis (*p*-value) is assessed to reject or accept a null hypothesis [152,153]. The null hypothesis is rejected if there is a significant difference between the models [69]. The Freidman test was used to evaluate the model performance without pairwise comparison. If the *p*-value is less than 0.05, the comparison results are not reliable [45]. The Wilcoxon sign-ranked test was used to check the statistical significance of systematic pairwise comparisons of models. The test results were evaluated based on *p*-values and *z*-values [58]. The null hypothesis is rejected if the *p*-value is less than 0.05 and the *z*-value exceeds the critical values of −1.96 and +1.96. In such a case, the performance of the models is deemed to be significantly different.

### 4.7. Factor Selection Using One-R Attribute Evaluation Technique

The selection of appropriate conditioning factors is perhaps the most important step in landslide prediction studies. Once chosen, the factors are used to create input data (training and testing datasets) for the machine learning models. A feature selection technique is employed to choose appropriate conditioning factors. It assesses the importance of each factor in predicting the final results and removes unimportant factors from the input space, thus preventing redundancy and reducing noise and over-fitting problems. In this way, the quality of input data is increased and the predictability of the landslide model is enhanced [154].

Many different feature selection methods have been proposed to select suitable factors for predictive models, including Information Gain [155], Forward Elimination [156], Backward Elimination [156], and One Rule Attribute Evaluation (ORAE) [157]. We employed ORAE, an effective filter selection method, [157] for this study. ORAE determines statistical correlations between an output variable and a set of selected input factors. One rule is separately created for each element in the training dataset, and the rule with the smallest error of detests is selected for modeling. In so doing, ORAE independently classifies all factors according to their importance to solve landslide prediction problems.

### 4.8. Summary of the Methodology Used in This Study

**Figure 5:**
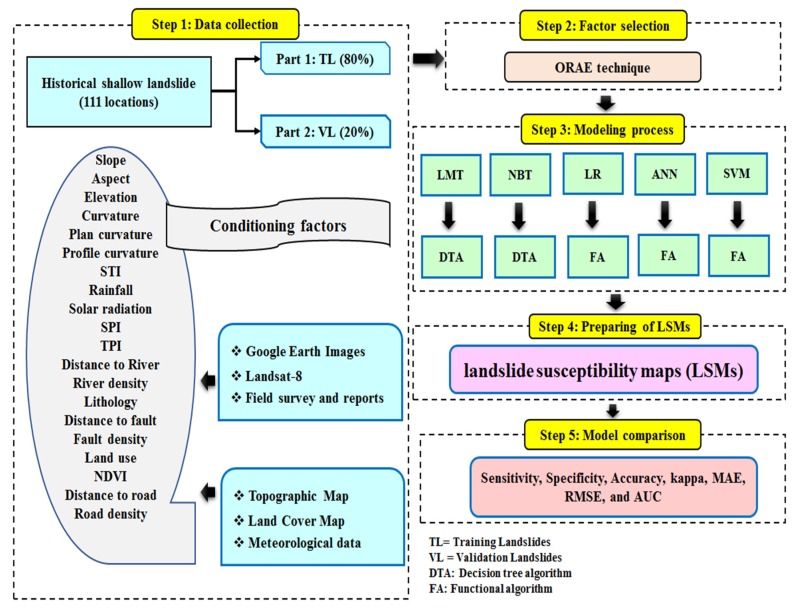
The flowchart of methodology used in this study.

Figure 5 provides a summary of the methodology used in our study. In this study, we prepared and used of the following steps for the modeling process and for preparing shallow landslide susceptibility maps:

Step 1: Data collection

We first created the landslide inventory map and selected possible landslide conditioning factors (Section 3.2, respectively, of this paper).

Step 2: Factor selection

We next used the ORAE feature selection technique to select the most important factors for landslide occurrence in the study area (Section 4.7).

Step 3: Modelling process

We next applied the LMT, NBT, LR, ANN, and SVM machine learning models using the most important factors determined in step 2 (Section 4.1, Section 4.2, Section 4.3, Section 4.4 and Section 4.5).

Step 4: Preparation of shallow landslide susceptibility maps

We applied each model to the training dataset and calculated a weight (shallow landslide susceptibility index) for each pixel of the study area. Based on these weights, we created shallow landslide susceptibility maps.

Step 5: Model comparison and validation

We used statistical indexes, namely sensitivity, specificity, accuracy, MAE, RMSE, and AUC, to check goodness-of-fit and prediction power using, respectively, the training and validation datasets (Section 4.6.1 and Section 4.6.2). Additionally, we tested the results using the Freidman and Wilcoxon statistical tests (Section 4.6.3).

## 5. Results and Analysis

### 5.1. Most Important Landslide Conditioning Factors

**Figure 6:**
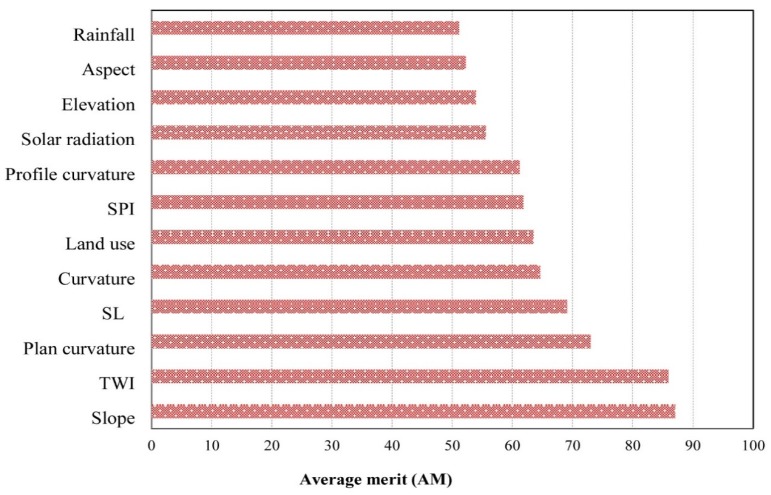
Average merit of shallow landslide factors calculated by the One Rule Attribute Evaluation (ORAE) method.

Using the ORAE method, we found that 12 of the 20 candidate conditioning factors had adequate predictive ability (AM > 0) to be used in modeling landslide susceptibility. The average merit, based on a 10-fold cross validation technique, is illustrated in Figure 6. Slope angle and TWI had the highest average merits (respectively, 87.08 and 85.96), followed by plan curvature (73.03), slope length (69.10), curvature (64.61), land use (63.48), SPI (61.80), profile curvature (61.24), solar radiation (55.62), elevation (53.93), aspect (52.25), and rainfall (51.12).

### 5.2. Model Performance and Analysis

The performances of the applied predictive models (LMT, NBT, LR, ANN, and SVM) were determined based on both the training and validation datasets (Table 1). In the case of the training dataset, the LMT model achieved the best goodness-of-fit, as quantified by MAE (0.207), RMSE (0.304), and AUC (0.944). The NBT and SVM models have the highest sensitivity (0.928), and the LR model has the best specificity (0.900) and accuracy (0.904). The NBT and SVM models have the best quality, with 92.8% of the landslide pixels properly assigned to the landslide class, followed by LR (90.9%), LMT (90.7%), and ANN (82.6%). The LR model has the highest specificity, with 90.0% of the no-landslide pixels properly classified in the no-landslide class. This model also has the best accuracy, with a 90.4% probability of properly categorized pixels, followed by the NBT and SVM (89.9%), LMT (89.3%), and ANN (83.7%) models.

For the validation dataset, both the LMT and LR models have the highest goodness-of-fit based on the MAE (0.216), RMSE (0.313 and 0.314), and AUC (0.936) measures, followed by the NBT model. The NBT model also has the best sensitivity, with 90.0% of the landslide pixels in the correct class. The LMT and LR models have the highest specificity (0.864), with 86.4% of non-landslide pixels in the right class. The next highest specificity (0.833) was obtained by the NBT and SVM models. In terms of accuracy, all of the predictive models except ANN have the same accuracy (0.864), indicating that the probability of pixels being correctly categorized is 86.4%. It is worth noting that ANN has the lowest values for sensitivity (0.762), specificity (0.739), and accuracy (0.750), and that SVM has the lowest goodness-of-fit based on the MAE (0.246), RMSE (0.369), and AUC (0.864) measures.

### 5.3. Development of Landslide Susceptibility Maps

**Figure 7:**
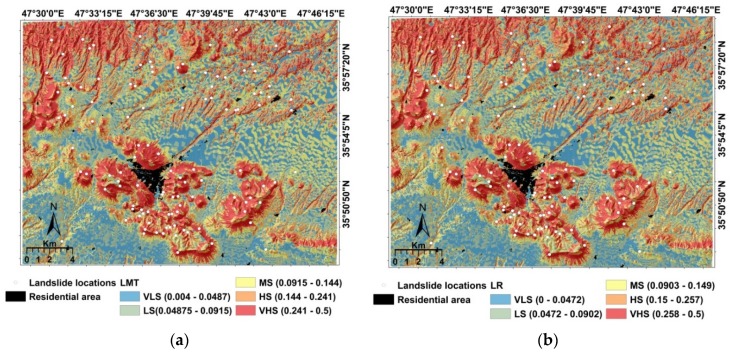

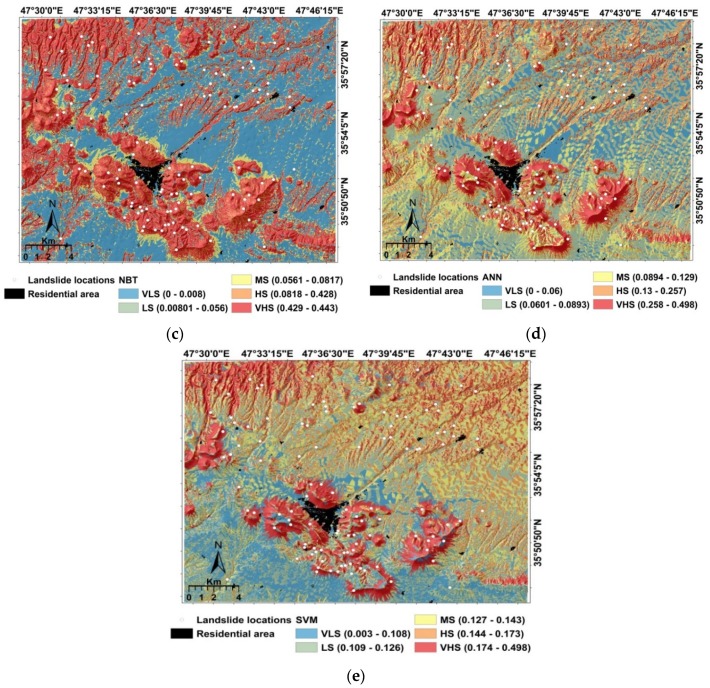
Landslide susceptibility maps: (**a**) Logistic Model Tree (LMT), (**b**) Logistic Regression (LR), (**c**) Naïve Bayes Tree (NBT), (**d**) ANN, and (**e**) SVM.

After developing the LMT, LR, NBT, ANN, and SVM models, we estimated landslide susceptibility indices (LSI) for each pixel in each model. LSIs were computed according to the probability distribution function of each model. In order to facilitate the visualization of the susceptibility models, we divided the indices into five susceptibility classes by the natural break method: very low (VLS), low (LS), moderate (MS), high (HS), and very high (VHS). Finally, we developed a susceptibility map for each of the five models (Figure 7). These maps consistently indicate that the south-central and northwestern parts of the study area, which are hilly and mountainous, are most susceptible to landslides.

### 5.4. Model Comparison and Validation

#### 5.4.1. ROC Curve

**Figure 8:**
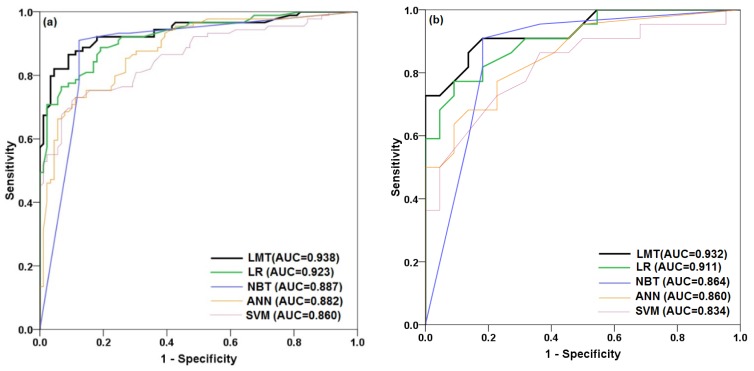
Receiver operating characteristic (ROC) curves and area under the receiver operatic characteristic curve (AUC) for the (**a**) training dataset and (**b**) validation dataset.

We evaluated the validity of the shallow landslide susceptibility maps based on the ROC curves and AUC values (Figure 8). The area under the curve for the training dataset is largest for the LMT model (0.938), followed by the LR (0.923), NBT (0.887), ANN (0.882), and SVM (0.860) models (Figure 8a). The area under the curve for the validation dataset is also highest for the LMT model (0.932), followed by the LR (0.911), NBT (0.864), ANN (0.860), and SVM (0.834) models (Figure 8b). These values suggest that the LMT model has the highest goodness-of-fit and prediction accuracy for the study area. Overall, the results indicate that the LMT classifier provides a higher quality landslide susceptibility model for the study area than the other machine learning methods.

#### 5.4.2. Wilcoxon Sign Rank Test

In addition to ROC, the statistical treatments of the five machine learning models were also analyzed by two well-known non-parametric statistical tests including the Friedman and Wilcoxon tests. As abovementioned, the null hypothesis is rejected if the significant level of a model is less than 0.05 (α = 0.05). The null hypothesis that there is no difference among the performances of the shallow landslide models at a significance level of α = 0.05 (5%) was rejected. In this case, it was concluded that two or more model are statistically different in terms of performance. The result of the Friedman test concluded that the significant value was less than 0.05 and hence the null hypothesis was rejected (true). The Friedman method, however, provides no information on pairwise comparison. The Wilcoxon test assessed systematic pairwise differences among the shallow landslide models and indicated significant differences among some of them (Table 2). There was no significant difference between the LMT and LR models, indicating that these two algorithms have similar predictive power. In contrast, the performances of the other models were significantly different from each other, and from the LMT and LR (Table 3).

## 6. Discussion

The ability to accurately estimate the sensitivity of terrain to landslides is an essential step in land-use planning [158]. Integration of advanced machine learning algorithms now allows researchers to develop landslide susceptibility models with high predictive capabilities. Land-use planners can use maps produced from these models to reduce landslide risk [159]. However, many different methods for modeling landslide sensitivity have been proposed, and the predictive accuracy of these methods continues to be debated [160]. The growth in computer processing power offers researchers new opportunities to compare models and evaluate their advantages and disadvantages.

Among the issues faced by researchers is the selection of appropriate landslide conditioning factors. Appropriate conditioning factors may differ from region to region, depending on geology, soils, topography, climate, and land use [161]. Thus, protocols must be developed to test the predictive ability of the entire suite of factors that are under consideration [162,163]. In this study, we prepared a landslide inventory map comprising 111 landslides and considered 20 conditioning factors. We used the ORAE method and the AM index to remove 12 of the 20 factors from the landslide modeling process. Slope has the highest average merits and is deemed to be the most critical factor in determining landslide susceptibility in the Bijar study area. Landslides in this area are most common on the steep, relatively wet slopes with sparse vegetation [54,164].

In this study, we compared the performance of five machine learning models: Logistic Model Tree, Logistic Regression, Naive Bayes Tree, Artificial Neural Network, and Support Vector Machine. All five models performed well, with classification accuracies >0.837 for the training dataset and 0.75 for the validation dataset. Model validation was performed using several statistical indices, for example accuracy and ROC. The LMT model provided the best balance of classification capability and performance. The LMT model uses leaf nodes and does not use constant values [161]. And according to Landwehr et al. [132], LMT is efficient in constructing logistic models at lower levels of the tree, rather than extending to lower levels models already established at higher levels. Moreover, the LMT algorithm applies cross-validation of LogitBoost iterations because training samples may be incorrectly modified. The LMT model was validated with statistical measures and ROC.

The LR, ANN, and SVM models require much more computer power and lengthy execution times. Typically, data are converted to ASCII format for statistical analysis and are later reformatted for integration into a GIS. In addition, processing of large amounts of data in the statistical package is more complicated for these three models [139,165,166]. A disadvantage of the NBT model is the assumption that it does not depend on the attribution. Research by Tien Bui et al. [22] suggests that this assumption may be incorrect, at least in the case of landslides.

We argue that the LMT model is an effective and simple tool for landslide susceptibility mapping. We acknowledge, however, that there is no consensus about the best method for modeling landslide susceptibility. In this paper, we compared five techniques: Logistic Model Tree, Logistic Regression, Naive Bayes Tree, Artificial Neural Network, and Support Vector Machine to evaluate landslide susceptibility in a semi-arid area in northwestern Iran. It is noted that all five methods perform well, but the LMT model is superior.

The proposed approach has advantages as well as limitations. The advantages are: (1) it has established and applied rules that are extractable and understandable; (2) it makes pair-wise comparisons; (3) it is structured to work quickly with large and complex datasets; (4) it can detect relationships and differences in subgroups and adjust for missing data; and (5) it does not rely on expert knowledge and experience to make decisions. However, it is limited by the available database and the choice of landslide conditioning factors. Small samples pose major obstacles. Future research should aim to find ways to reduce the small dataset problem, for example by replacing landslide points with landslide polygons which would significantly increase the number of pixels and improve the quality of models.

## 7. Conclusions

Accurate landslide susceptibility maps assist land-use planners and government officials to reduce loss of life and damage from slope failures. In this study, we prepared landslide susceptibility maps for the area around Bijar City, Iran, using five soft-computing benchmark algorithms: LMT, LR, NBT, ANN, and SVM. Our database comprised 111 shallow landslides. We divided the landslides into training and prediction groups and selected 20 landslide conditioning factors for modeling based on the Information Gain Ratio technique. All data were elaborated in a GIS environment. We determined that slope angle and the topographic wetness index are the most important factors for shallow landslide occurrence in the study area. The hilly and mountainous parts of the study area have a higher likelihood of shallow landslides, especially if their soils are saturated.

Although all five machine learning models performed well, the LMT model outperformed the others. It thus has considerable promise as a tool for mapping shallow landslide susceptibility in other semi-arid regions with similar topography, geology, and climate. We recommend it as a tool to help planners, managers, and government agencies mitigate landslide hazards. The LR model outperformed the NBT, ANN, and SVM models, thus we consider it to be a trustworthy model for identifying shallow landslide-prone areas in semi-arid environments.

A long-term goal of landslide researchers is the development of protocols for producing accurate landslide susceptibility maps. Many hurdles remain before this is possible, including limitations in available data, unknown factors, and known factors that are dynamic in nature (e.g., temporal changes in climate and land use). Thus, much more research is needed, and we advise caution in generalizing results in one area to others.

## Figures and Tables

**Table 1:** Model performances of the applied data-mining approaches for the training and validation datasets.

Parameters	LMT		NBT		LR		ANN		SVM	
	T *	V *	T	V	T	V	T	V	T	V
**True positive**	78	19	77	18	80	19	76	16	77	18
**True negative**	81	19	83	20	81	19	73	17	83	20
**False positive**	11	3	12	4	9	3	13	6	12	4
**False negative**	8	3	6	2	8	3	16	5	6	2
**Sensitivity (%)**	0.907	0.864	0.928	0.900	0.909	0.864	0.826	0.762	0.928	0.900
**Specificity (%)**	0.880	0.864	0.874	0.833	0.900	0.864	0.849	0.739	0.874	0.833
**Accuracy (%)**	0.893	0.864	0.899	0.864	0.904	0.864	0.837	0.750	0.899	0.864
**MAE**	0.207	0.216	0.225	0.225	0.213	0.216	0.241	0.235	0.223	0.246
**RMSE**	0.304	0.313	0.319	0.341	0.311	0.314	0.349	0.358	0.318	0.369
**AUC**	0.944	0.936	0.918	0.874	0.939	0.936	0.911	0.871	0.899	0.864

T *: Training, V *: Validation.

**Table 2:** Performance of the five landslide machine learning models using Wilcoxon signed-rank test (two-tailed).

No.	Landslide Model	Mean Rank	χ2	*p*-Value
1	LMT	2.80	557.912	0.000
2	LR	2.93		
3	NBT	2.88		
4	ANN	3.07		
5	SVM	2.32		

**Table 3:** Performance of the five landslide machine learning models using the Wilcoxon signed-rank test (two-tailed).

No.	Pairwise Comparison	Number of Positive Differences	Number of Negative Differences	*z*-Value	*p*-Value	Significance
**1**	**LMT vs. LR**	60	50	−1.536	0.125	No
**2**	**LMT vs. NBT**	83	27	−5.590	0.000	Yes
**3**	**LMT vs. ANN**	62	46	−0.878	0.080	Yes
**4**	**LMT vs. SVM**	36	74	−3.677	0.000	Yes
**5**	**LR vs. NBT**	82	29	−5.589	0.000	Yes
**6**	**LR vs. ANN**	61	49	−0.605	0.015	Yes
**7**	**LR vs. SVM**	35	75	−4.081	0.000	Yes
**8**	**NBT vs. ANN**	36	73	−3.958	0.000	Yes
**9**	**NBT vs. SVM**	30	80	−5.711	0.000	Yes
**10**	**ANN vs. SVM**	43	67	−3.140	0.002	Yes

Note: The standard *p*-value is 0.05.
